# Nuclear Lamins: Key Proteins for Embryonic Development

**DOI:** 10.3390/biology11020198

**Published:** 2022-01-27

**Authors:** Jasper Chrysolite Paul, Helena Fulka

**Affiliations:** Institute of Experimental Medicine, Czech Academy of Sciences, Vídeňská 1083, 142 20 Prague, Czech Republic; paul.jasper@iem.cas.cz

**Keywords:** nuclear lamins, laminopathies, preimplantation embryo, development, maternal factors

## Abstract

**Simple Summary:**

The biology of a multicellular organism is extremely complex, leaving behind a realm of compound yet systematic mechanisms still to be unraveled. The nucleus is a vital cellular organelle adapted to storing and regulating the hereditary genetic information. Dysregulation of the nucleus can have profound effects on the physiology and viability of cells. This becomes extremely significant in the context of development, where the whole organism arises from a single cell, the zygote. Therefore, even a mild aberration at this stage can have profound effects on the whole organism. However, studying the function of individual nuclear components at this point is exceptionally complicated because this phase is inherently under the control of maternal factors stored in the female germ cell, the egg. Here, we focus on the lamins, as essential nuclear components, and summarize the current knowledge of their role in development. Although scientists encounter challenges working with these miniscule yet key proteins, the demand to know more is increasing gradually due to the mutations caused in lamins leading to irreversible phenotypic conditions in humans.

**Abstract:**

Lamins are essential components of the nuclear envelope and have been studied for decades due to their involvement in several devastating human diseases, the laminopathies. Despite intensive research, the molecular basis behind the disease state remains mostly unclear with a number of conflicting results regarding the different cellular functions of nuclear lamins being published. The field of developmental biology is no exception. Across model organisms, the types of lamins present in early mammalian development have been contradictory over the years. Due to the long half-life of the lamin proteins, which is a maternal factor that gets carried over to the zygote after fertilization, investigators are posed with challenges to dive into the functional aspects and significance of lamins in development. Due to these technical limitations, the role of lamins in early mammalian embryos is virtually unexplored. This review aims in converging results that were obtained so far in addition to the complex functions that ceases if lamins are mutated.

## 1. Introduction

The majority of the eukaryotic cells, except for the red blood cells, have a membrane bound organelle, the nucleus. Occupying a variable proportion of the total cellular volume in different cell types and significantly known as the storehouse of hereditary genetic information, the nucleus regulates replication and subsequent transcription of DNA [1,2]. The exceptional feature of the metazoan cell nucleus is the presence of a nuclear envelope (NE), which demarcates the cytoplasm from the nucleoplasm. The nuclear envelope plays a vital role in regulating gene expression [3], chromatin reorganization [4], in addition to nuclear integrity [5]. All of these roles are exerted by the combination of selective nuclear transport of materials such as proteins or RNAs across the NE and by providing structural and mechanical support for setting up the 3D organization of the nucleus and chromatin. The correct NE function is extremely important in the context of very early embryos as the whole organism arises from only a single cell, the zygote. Therefore, every dysregulation at this stage can have a profound effect, not only on the somatic cells of the new organism, but also potentially impact the next generation if the primordial germ cells, the predecessors of gametes, are affected. While the nuclear envelope has been shown to be a dynamic structure with respect to its composition in somatic cells [6,7] (for review see [8]), possibly reflecting the immediate physiological state, the situation in early embryos is different. Parental gametes, unlike their somatic counterparts, are transcriptionally inactive and are, therefore, reliant on the maternal pool with different resources, both proteins and mRNA, which are accumulated by oocytes prior to fertilization. Since the components of the NE are directly inherited, the embryos most likely possess limited options to regulate NE composition and possibly functional correction, should the situation demand. For this reason, a precise number of maternal factors and their faithful regulation during the pronuclear formation and assembly of the NE are vital for a successful development and execution of the developmental program. Although the overall structure of the nuclear envelope is immensely complex, in this review, we will focus specifically on the nuclear lamina, a proteinaceous structure of the inner NE. Over the years, NE components and its structural proteins, the nuclear lamins, have received considerable attention due to their role in several overwhelming human diseases as well as their link to cellular differentiation.

Although our view of the role of nuclear lamins in development has been traditionally shaped in conjunction with the different disease states, in light of recent studies we might have to reconsider their biological roles as these might be far more complex than previously anticipated.

**Figure 1:**
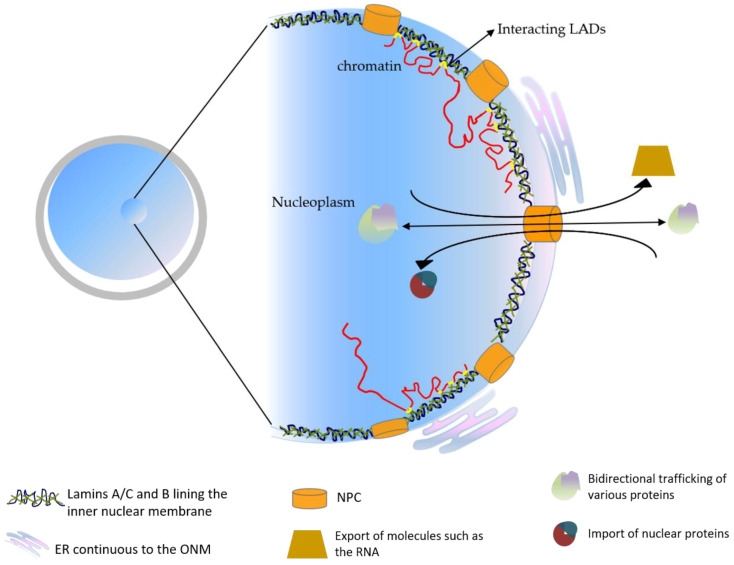
The schematic representation of the basic nuclear organization.

The nuclear envelope consists of a lipid bilayer. Very early electron microscopy showed that the structure of the outer nuclear membrane (ONM) as being continuous with the endoplasmic reticulum [9]. Sandwiched between the inner nuclear membrane (INM) and the peripheral chromatin inside of the nucleus is a protein meshwork called the nuclear lamina [10]. This protein meshwork was proposed to not only provide mechanical support, but also to be a major component to promote chromatin anchoring to the nuclear pore complex that spans the ONM and INM and facilitates the communication between the nuclear interior and the cytoplasm [11]. The key component of the proteinaceous layer called the nuclear lamina, coating the INM, is in most metazoan cells, the lamins [12] (Figure 1).

The nuclear lamins have historically received substantial attention due to the number of mutations (as of 31 January 2020, nearly 500 different mutations have been identified in the human *LMNA* gene, http://www.umd.be/LMNA/ accessed on 28 December 2021) and their association with several devastating human diseases called laminopathies [13,14,15]. Due to the extensive research of lamin phenotypes, their proposed functions range from a direct regulation of gene activity [16,17] and/or locking in the differentiated state in development [18], through their role in DNA replication and repair to preserving mitotic spindle structure and promoting chromosome segregation fidelity [19,20]. However, as we will describe in more detail in latter parts of this review, the overall picture might be skewed by the different experimental models and systems used by individual research groups and not all the observed phenotypes and functions were corroborated when such experimental model was exchanged. Therefore, when describing the lamin function(s), the biological context seems crucial. Next, we will describe the structure of these proteins, their basic biology and their how mutations manifest in humans.

## 2. Structure of Lamins

Nuclear laminae are type V intermediate filaments (IF) that are evolutionarily conserved in eukaryotes [21]. Restricted to the animal kingdom, electron microscopy of isolated plant nucleo-skeleton show structures similar to that of the metazoan lamina. Although plants lack lamins and genes that encode for the lamin-binding proteins [22], the function is substituted by the nuclear matrix constituent proteins (NMCP) [23]. The presence of an equivalent dense proteinaceous structure lining the INM indicates its wider importance and a possible common function in several organisms.

There are two main types of lamins based on their domain structures, A-type lamins and B-type lamins (lamin A and lamin B, respectively). A- type lamins are coded by the single gene, *Lmna*. Alternate splicing of the *Lmna* gene in the mammalian system leads to the production of lamin A and lamin C. The gene *Lmna* also encodes for a less abundant, somatic cell isoform of lamin called lamin AΔ10 [24]. Differential splicing of the A-type lamins in mouse pachytene spermatocytes [25] and in the early stages of different mammalian oocytes [26] produces lamin C2, showing similarities with lamin C. The B- type lamins are of two types: *Lmnb1* and *Lmnb2*. While *Lmnb1* encodes for a single type of lamin protein, lamin B1, *Lmnb2* encodes for lamin B2 and a lamin B3, which is produced by the differential splicing and restricted to the mouse spermatocytes [27]. Unlike lamin B, which are expressed in all tissues and are ancestrally conserved [28], lamin A was described to be expressed only in terminally differentiated tissues and dispensable for mouse development [29]. In rat liver nuclei, the lamina was described as a 150 Å thick proteinaceous layer underlying the inner nuclear membrane [30], and associated with the nuclear pore complex. In 1993, the first reported *Caenorhabditis elegans* nuclear lamins [31] formed intermediate filament-like structures which were approximately 10 nm long [32]. However, analysis of the lamin filaments in other model organisms showed their relatively variable properties. In *Xenopus*, the average lamin B filament length was 15 nm, whereas exogenously produced lamin A filaments were shorter but thicker [33]. A recent very detailed study using mouse cells described the presence of filaments of a highly variable length ranging from 50 nm to 2700 nm [34]. Irrespective of the variations described, the ability to polymerize and form filaments lies at the heart of the lamina formation.

Identical to most intermediate filaments [11], the conserved structure of lamins consist of an elongated central α-helical coiled rod domain flanked by variable amino -terminal head and carboxy- terminal tail domains [35] with a relative molecular mass (M_r_) between 60,000 to 75,000 [12]. At the carboxyl end, the lamins contain a sequence CaaX motif which provide site modifications such as isoprenylation and methylation, which have been shown to be important to associate the lamins to the inner nuclear membrane [36] (p. 2). The absence of codons for 82 amino acids including the CaaX motif, and with a striking homology rises another variant of lamin A, is lamin C [11]. Characteristic to all lamin types, between the C-terminal and the rod domain are the nuclear localization signal sequences (NLS) which is vital for nuclear transport [37,38]. The interaction of lamins and the nuclear transport machinery might be more important than just providing the means of their nuclear import. Interestingly, the level of the nuclear transport factor importin α was suggested to influence the lamin assembly possibly by preventing their self-association as well as by modulating the interaction with their binding partners [37].

## 3. The Consequence of Lamin Genes Disruption

The formation of this proteinaceous meshwork begins by the organization of lamin dimers at the interphase of chromatin and the nucleoplasmic surface of the inner nuclear membrane [12]. Initially suggested to provide only nuclear integrity [39], the assembly of lamins are key to the diverse functions such as DNA replication, DNA repair, chromatin anchorage, and even gene regulation, therefore the mutations in genes encoding lamins cause serious malfunctioning in humans, leading to a wide range of disease conditions [40]. Studies have shown the mutation in the *LMNA* gene encoding for lamin A/C to be detrimental in affecting the striated muscles to cause diseases such as Emery–Dreifuss muscular dystrophy [41] and postnatal growth retardation [42], inheritable disorders such as limb-girdle muscular dystrophy [43], and dilated cardiomyopathy leading to abnormal postnatal growth retardation and death of newborns [44]. In addition to the muscular dystrophies caused by the mutation of lamin A/C, there are reports that involve bone tissue, adipose tissue, the peripheral nervous tissues, and also a syndrome called the Hutchinson–Gilford progeria syndrome (HGPS) (Refer Table 1 below).

Interestingly, although a number of mutations in the *LMNA* gene have been described (see *Introduction*), practically no laminopathies linked to B-type lamins (*LMNB1/LMNB2* mutations) have been reported to date with very few exceptions presented in Table 1. For this reason, the general notion is that mutations in the genes encoding for B-type lamins, or their loss, are developmentally lethal and that lamins B1/B2 might be essential at the cellular level, i.e., necessary for cell viability. This conception is in agreement with early developmental studies, showing that while LMNA was detected only in some cells and in conjunction with a more advanced differentiation state, the B-type lamins were present in all probed cell types irrespective of their differentiation state [60,61]. Indeed, fitting with the general view, knocking down the *LMNA* mRNA by siRNA in HeLa cells has been described to have no effect, whereas both *LMNB1* and *LMNB2* were shown to arrest the cells and to cause cellular death [62]. Although widely accepted, this view has been strongly challenged by more recent studies produced using animal models instead of transformed cells in culture [62]. Both *Lmnb1-* or *Lmnb2*-deficient animals progressed through development, but died shortly after birth [63,64]. Although these results indicate the non-essentiality of B-type lamins, it was not possible to exclude that, under these conditions, the second lamin B gene partially rescued the phenotype. This, in turn, indicated a certain level of B-type lamin redundancy. Nevertheless, this possibility has been partially addressed by Yang and colleagues who produced mice with a double deletion of both *Lmnb1* and *Lmnb2* in keratinocytes [65]. As reported by the authors, the mice survived for an extended periods (over two years of age) and were grossly normal showing that, at least in this specific cell type, B-type lamins are indeed not essential. Practically, the same conclusion has been reached even at the organismal level when double *Lmnb1*/*Lmnb2* knockout mice were produced [66]. As reiterated in the aforementioned studies, postnatal death of these animals was caused due to lack of breathing. Although these animals had all essential internal organs, more detailed analysis revealed that the overall size was slightly smaller when compared to their littermates [66]. In summary, these animal models provide practically no support for the generally accepted notion of lamin B essentiality.

Assuming that the mouse models developmentally reflect the situation in humans, one would expect at least some record of B-type lamin mutations in human patients, stillborn babies, or in relation to miscarriages, as the redundancy between the lamin B gene products could be excluded. Still, practically no lamin B mutations were clinically reported. There might be essentially two explanations: firstly, either the mouse model is developmentally unique or additional mammalian models would bring different results, and secondly, lamins are only essential immediately after the post fertilization period. Undoubtedly, the second explanation cannot be strictly ruled out since the various homozygous knockout animals used for the phenotype analysis, which are not viable after birth, were produced by the breeding of the heterozygous parents. Therefore, the egg, which will give rise to the individual homozygous knockout embryos, will be pre-loaded with both maternal proteins and their respective mRNAs. Effective manifestation of gene disruption can only begin when the maternal stores are depleted. This could potentially mask the early phenotype. This makes the analysis of the role nuclear lamins in the early post-fertilization phase extremely difficult. Next, we will focus on female germ cells and early embryos and summarize the results on the role of nuclear lamins at these stages of development.

## 4. The Cellular Functions of the Lamins in Development

Historically, the system that was used to investigate the function of nuclear lamins which also greatly shaped our perception of their cellular function, were the *Xenopus* egg extracts and in vitro assembly of nuclei. Oocytes of *Xenopus laevis* were found to exhibit only one type of lamin—the lamin L111 (laminB3 L homeolog, lmnb3.L - https://www.ncbi.nlm.nih.gov/gene/397910; accessed on 28 December 2021). By contrast, somatic cells, such as erythrocytes, were found to express additional types: L1 (lamin B1, lmnb1; https://www.ncbi.nlm.nih.gov/gene/394806; accessed on 28 December 2021) and L11 (lamin B2, lmnb2; https://www.ncbi.nlm.nih.gov/gene/100038111; accessed on 28 December 2021) [67], and differentiated cells such as myocytes and neurons expressed all three types: L1, L11, and L111 [68]. The presence of one major type of lamin protein, the large size of the *Xenopus* eggs and embryos, as well as their abundance and the relative ease of manipulation make the system of extracts ideal to elucidate the mechanism of nuclear assembly and function of different nuclear components.

In 1990, Newport, Wilson and Dunphy showed that the lamin LIII is not essential for the nucleus formation, but the artificially formed nuclei produced in lamin LIII immunodepleted extracts were fragile and failed to expand [69]. Quite interestingly, nuclei formed in these extracts also failed to replicate the DNA [69]. A similar observation was further made in 1991 by Meier and colleagues [70], who also failed to detect the incorporation of radiolabeled isotopes to nuclei formed from demembranated sperm heads in extracts where LIII was blocked by an antibody. The lack of DNA replication is somewhat puzzling in context of the lamin B knockout animal models described above. However, since lamin LIII, being the predominant lamin type in this system, was blocked or depleted, it might be possible that the presence of at least one type of lamin, irrespective of whether A- or B-type, is crucial to ensure the DNA replication capacity. However, this is contradicted by the study where the lamin triple-knockout embryonic stem cells were generated and analyzed [29]. To maintain cells in culture, replication of their DNA is vital. Therefore, it is unknown whether the observed replication block can be attributed specifically to the lamin LIII. Another unexpected role of the *Xenopus* oocyte lamin is its participation in the spindle assembly by forming a so-called spindle matrix [71]. The spindle matrix and spindle could be disrupted by again immunodepleting the *Xenopus* M-phase extracts with either a monoclonal or a polyclonal antibody against the oocyte-specific lamin type. 

Both replication and spindle assembly are indeed vital cellular functions, however, whether the results obtained in *Xenopus* egg extracts can be extrapolated to other tissues, other model organisms or the remaining lamin protein types is unclear. On the one hand, the structural comparison between the naturally occurring A- type lamins to the B- type lamins in *Xenopus* revealed that the B-type lamins were relatively conserved and ancestral to the A- type lamins that were produced due to exon shuffling among the same species [28], which would indicate that the function might be shared among the different lamin types. On the other hand, there are various results showing that individual lamins have gene-specific roles.

The situation in mammalian oocytes and eggs are much more intricate. The information available comes mostly from the murine [72,73], bovine, and porcine oocytes and eggs [74,75]. Although the results on the presence of different lamin types tend to vary, which can probably be attributed to the different antibodies used by the authors and a well-known property of epitope masking of the nuclear structural proteins [76], we may presume that all lamin types are present in oocytes and eggs. Indeed, a more recent proteomic analysis supports this conclusion [77,78]. Because the oocyte nuclear proteins serve as building blocks which is utilized by the early embryo prior to its own genome activation, it is likely that the maternally inherited lamins directly participate in the parental pronucleus formation [79]. However, it cannot be strictly ruled out at present that maternal lamins are degraded during the meiotic maturation and/or early post-fertilization period and synthesized de novo based on maternal mRNA. Only later, together with the major embryonic genome activation, can we expect a more dynamic regulation of the nuclear lamina in embryos. Concurrently, in 1988, Houliston et al. [80] described that all three lamins, lamin A/C and lamin B, were present in the mouse pre-implantation embryos, right from the fertilized egg to the blastocysts. Specifically, lamin A was more predominantly found to be expressed in the oocytes or unfertilized eggs than blastocysts and other mouse cell types indicating a gradual depletion of the maternal lamin A. Lamin B was detected in 8-cell stage and blastocysts [80]. However, in this case a straight conclusion on the re-expression of lamin B cannot be made due to the limited embryonic stages analyzed, since its presence can be theoretically caused by a mere unmasking of the maternal protein. Nevertheless, essentially the same results were obtained by Schatten et al. [73] and Maul and colleagues in 1987 [81]. Taken together, these studies suggest that the specific lamins in the later stages of embryo development are not, at least to some degree, from the maternal pool of reserves. 

In the context of the above mentioned general view of the role of lamins in development, these findings, to a great extent, contradict the widely accepted opinion that the presence of lamin A/C only appears with an advanced differentiation state [60]: the zygote is a quintessential totipotent cell which has the ability to give rise to all cells of the body, and therefore represents the least differentiated state while still having a noticeable amount of this lamin type. Furthermore, the spindle localization as described in *Xenopus* was also not confirmed in mammals [73,74,75,82]. Therefore, the functional significance of different lamin types in early mammalian embryos remain elusive.

## 5. Lamin Function in Early Mammalian Embryos

**Figure 2:**
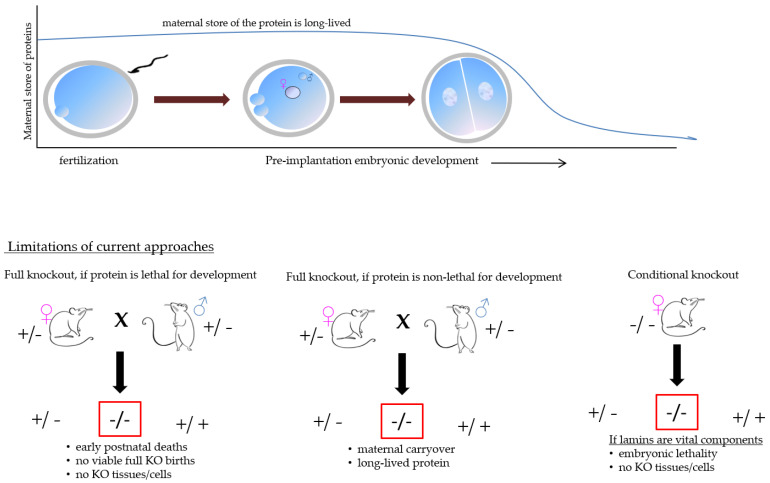
The pictorial representation of the technical challenges that could be encountered.

The very biology of mammalian fertilization, i.e., the presence of maternal materials, the limited availability of mammalian oocytes and embryos in combination with the long half-life of lamin proteins make the analysis of putative lamin functions extremely difficult, see Figure 2. More specifically, the widely popular approaches used to study the gene function in mammalian embryos, i.e., siRNA or morpholino oligo injection, will likely not produce a change in the protein level rapidly enough (HF, personal experience). For these reasons, very few options remain.

In somatic cells, one of the functions of nuclear lamina is to participate in the 3D organization of chromatin, reviewed in [83], which, in turn, can lead to changes in gene expression, for review see [84]. Based on a differential staining of chromosomes, between 1928 and 1935, it was Emil Heitz who coined the term heterochromatin and euchromatin. Through the years of constant investigation of the expression studies and the compartmentalization, it was found that heterochromatin is essential to transcriptionally regulate genes according to specific cell type by genes repression [85]. In somatic cells, the nuclear periphery and the nucleolus are typically the sites where heterochromatin localizes and the peripheral positioning often, but not always, correlates with the inactivity of the DNA sequences, for review see [86,87].

Advanced technique such as the high throughput chromatin conformation capture-based (Hi-C) assay coupled with DNA adenine methyltransferase identification (DamID) can be used to identify protein-DNA interactions. By engineering a specific fusion construct of Dam and a protein of interest, one can identify the DNA sequences which come into close contact with this exogenous fusion protein [88]. Dam is a bacterial (*Escherichia coli*) enzyme capable of introducing a stable methylation at the 6th position of Adenine giving rise to N6-methyladenine (m6A), a DNA modification thought to be absent in mammals [89]. When lamin-Dam fusion protein is exogenously introduced into cells, DNA sequences in contact with the nuclear lamina can be probed and analyzed [90,91,92]. Although very powerful, there are some limitations of the DamID method: the level of the exogenously introduced fusion protein must not interfere with a normal cellular function, and only DNA regions harboring the Dam methyltransferase recognition sequence (GATC) will be labelled [89]. Unfortunately, this motif is not evenly distributed throughout the mammalian genome. Therefore, sequences such as the satellites, the basic units of centromeres, pericentric chromatin, and telomeres—the prototypical examples of sequences associated with the nuclear lamina—fall through the cracks [93]. Next, the m6A modification of DNA is not as scarce as previously thought [94], and seems to be very abundant in embryos, including mammals [95,96]. Nevertheless, using this technique, a more detailed picture of the individual elements and genes in close contact with the nuclear lamina has been elucidated [92,97]. Complex compartmentalization of heterochromatin with respect to their nuclear organization leads to genome stability. Errors in organization leads to irreversible repair, and disease progression [98,99,100]. 

Nevertheless, when applied to early embryos, it has been shown that LADs are established de novo in the post-fertilization period and asymmetrically between the two parental genomes [101]. This is not too surprising, given the distinct epigenetic remodeling of the parental genomes after fertilization. While the maternal (oocyte) genome exhibits somatic-like features, the paternal genome undergoes much more extensive changes. In order for the male pronucleus to be formed, the paternal protamines, which organizes DNA in the sperm to a near crystalline state, needs to be removed and replaced by histones originating from the oocyte cytoplasm, for review see [102,103]. By contrast, the maternal genome is organized by histones throughout the whole oogenesis. 

In line with the general mechanism of histone incorporation, histones associated with the paternal DNA are mostly acetylated [104]. The methylation marks are established only later on, following replication (for review, see [105,106]). Although the first embryonic replication is essential for further embryonic development, it seems to be dispensable for the establishment of parental LADs [101]. Instead, the paternal LADs were shown to be dependent on the methylation of H3K4. The link between the epigenetic status of the parental genomes and the LAD establishment seems to be further supported by the convergence of parental LADs no earlier than the eight-cell embryonic stage [101], when the parental epigenetic asymmetry is equalized [107] (p. 1). Although the study by Borsos and colleagues [101] brings interesting insight into the lamina-DNA interaction establishment following fertilization, several important questions arise. 

The most important question is how faithfully does the analysis recapitulate the in vivo state? Here, it is important to bear in mind that certain sequences will not be captured by the DamID technique, as already mentioned [93]. However, in very early embryos, rather than the nuclear lamina, the major organizing structure seems to be the nucleolus precursor bodies, the embryonic equivalents of nucleoli [108]. Therefore, the inherent inability of the DamID to detect these sequences might not be as problematic as one might initially expect. However, there are additional caveats, such as lamin B1 not being the only protein interacting with and organizing specific sequences. For example, at least in some cells, the heterochromatin tethering to the nuclear lamina was shown to be dependent on lamin A and lamin B receptor [109]. Moreover, abrogating the DNA tethering led to changes in gene expression. In the context of the aforementioned study, it is currently unclear whether the association of specific sequences with lamin B1 in embryos has a functional consequence. The overexpression of *Kdm5b* by the mRNA injection leads to a general decrease of the H3K4me3 mark, which will likely have a wider consequence that simply overrules the LAD establishment in the paternal genome [101]. Unfortunately, no further functional and developmental data can be obtained under the described experimental setup. Irrespective of the technical limitations, the study brings interesting insights into how LADs are established post fertilization. 

## 6. Future Directions

Although the nuclear lamins have been long recognised for their importance in normal cellular biology, their specific properties and current technical limitations leave their distinct roles in early development largely unrecognised. Understanding their precise biology, developmental roles, their interaction partners, and interacting DNA sequences of nuclear lamins promise to bring key insights, which might lead to alleviating the dramatic phenotypes associated, namely, with the lamin A/C mutations. As a proof of this concept, mutation studies that induce Emery–Dreifuss muscular dystrophy (EDMD) conducted in C. elegans lamin (LMN-Y59C) coupled with the DamID identification of lamina associated chromatin showed that ablation of a protein that anchors H3K9- methylated chromatin, CEC-4, in the LMN-Y59C condition was able to reduce the disease phenotype and improve mobility of the worms [110]. Although mammals, including humans, are much more complex than *C. elegans*, a similar strategy of deleting a lamin A/C interaction partner was shown to ameliorate the phenotype of *Lmna* ablation in mice [111].

In contrast to the situation with Lamin A/C, the near total absence of clinical phenotypes associated with mutations or absence of lamin B1/B2 in humans is, to say the least, puzzling. Whether this absence is given by the redundancy between the lamin B genes or the prenatal severity of the phenotype is currently anonymous. One of the major limitations faced with investigating the role of lamins in the very early stages of mammalian development is the lack of appropriate tools. For this reason, the function of specific lamin genes and their products are virtually unprobed. Since the ablation of lamins, which are expressed in the early stages of egg development, could cause nuclear irregularity and chromatin instability already in the female germ cells, conditional knock- out or homozygous knock out studies can be challenging. Overall, mutation studies in vivo seem to be highly challenging as the ablation of vital genes such as the lamins may never lead to production of preimplantation embryos at all. At the same time, many of the lamin-associated phenotypes described in tissue cultures were recapitulated. Due to these limitations, novel and more refined strategies are considered necessary.

## Figures and Tables

**Table 1:** Some of the defects and irregularities caused by the mutations in lamins.

Disease	Lamin Mutations
Emery-Dreifuss muscular dystrophy (EDMD)	X- linked recessive disorder, Emerin (STA) mutation [45] Mutation in Lamin A/C produced by the alternate splicing of *LMNA* gene [46]
Mandibuloacral dysplasia and partial lipodystrophy	Homozygous missense mutation, Arg527His, in *LMNA* gene. Muation in ZMPSTE24 [47]
Mandibuloacral dysplasia with type A lipodystrophy (MADA)	Homozygous mutation in R527H in the *LMNA* gene [48]
Restrictive dermopathy (RD)	Dominant **de novo** *LMNA* mutations, recessive ZMPSTE24 [FACE-1 in humans] mutations (either homozygous or heterozygous), both within exon 9 [49] RD with ZMPSTE4 mutation—a complete absence of Lamin A protein—also a factor to identify lethal neonatal laminopathy
Hutchinson- Gilford progeria syndrome (HGPS)	Single point mutation in *LMNA* gene causing production of permanently farnesylated mutant Lamin A protein, progerin [50] (p. 22) Recurrent **de novo** single-base substitution within exon 11 of *LMNA* [51]
Limb Girdle muscular dystrophy type 1B (LGMD1B)	Mutation linked to the chromosome 1q11-q21 of *LMNA* gene [43]
Dilated cardiomyopathy (DCM)	R89L, 959delT, R337H, S573L mutation in *LMNA* [52,53]
Autosomal recessive axonal Charcot-Marie-Tooth type 2 (CMT2)	R298C mutation in lamin A/C [54]
Dunnigan type familial partial lipodystrophy (FPLD)	R482Q mutation in lamin A/C, mutation in the gene mapped to chromosome 1q21-22 encoding for the *LMNA* gene [55]
Adult autosomal dominant leukodystrophy (movement disorder)	Associated with increase or accumulation of lamin B1 [56]
Primary microcephaly (neuro—developmental disorder)	Heterozygous dominant pathogenic variants in both lamin B1 and lamin B2 [57]
Progressive myoclonus epilepsy including the early identification of ataxia	Rare and novel homozygous missense p.His157Tyr mutation in the alpha- helical rod of the lamin B2 protein [58]
Acquired partial lipodystrophy (APL)	Mutation in the *LMNB2* gene on 19p13.3 might be the cause of this disease [59]

## Data Availability

Not applicable.
